# Ratio dependence in small number discrimination is affected by the experimental procedure

**DOI:** 10.3389/fpsyg.2015.01649

**Published:** 2015-10-29

**Authors:** Christian Agrillo, Laura Piffer, Angelo Bisazza, Brian Butterworth

**Affiliations:** ^1^Department of General Psychology, University of PadovaPadova, Italy; ^2^Cognitive Neuroscience Center, University of PadovaPadova, Italy; ^3^Institute of Cognitive Neuroscience, University College LondonLondon, UK; ^4^National Chengchi UniversityTaipei, Taiwan; ^5^School of Psychological Sciences, University of Melbourne, MelbourneVIC, Australia

**Keywords:** OTS, ANS, subitizing, numerical cognition, non-verbal cognitive systems

## Abstract

Adults, infants and some non-human animals share an approximate number system (ANS) to estimate numerical quantities, and are supposed to share a second, ‘object-tracking,’ system (OTS) that supports the precise representation of a small number of items (up to 3 or 4). In relative numerosity judgments, accuracy depends on the ratio of the two numerosities (Weber’s Law) for numerosities >4 (the typical ANS range), while for numerosities ≤4 (OTS range) there is usually no ratio effect. However, recent studies have found evidence for ratio effects for small numerosities, challenging the idea that the OTS might be involved for small number discrimination. Here we tested the hypothesis that the lack of ratio effect in the numbers 1–4 is largely dependent on the type of stimulus presentation. We investigated relative numerosity judgments in college students using three different procedures: a simultaneous presentation of intermingled and separate groups of dots in separate experiments, and a further experiment with sequential presentation. As predicted, in the large number range, ratio dependence was observed in all tasks. By contrast, in the small number range, ratio insensitivity was found in one task (sequential presentation). In a fourth experiment, we showed that the presence of intermingled distractors elicited a ratio effect, while easily distinguishable distractors did not. As the different ratio sensitivity for small and large numbers has been often interpreted in terms of the activation of the OTS and ANS, our results suggest that numbers 1–4 may be represented by both numerical systems and that the experimental context, such as the presence/absence of task-irrelevant items in the visual field, would determine which system is activated.

## Introduction

A large body of experimental evidence collected in cultural, developmental, comparative and cognitive psychology supports the existence of numerical systems that are not related to language and culture. For example, newborns are able to discriminate between 4 and 12 objects (Izard et al., 2009), and 6-month old infants are able to distinguish between 6 and 12 objects well before the emergence of language (Xu and Spelke, 2000). Sophisticated numerical abilities have been described in animal species that lack a symbolic language, such as rodents and fish (reviewed in Agrillo and Bisazza, 2014). Pre-verbal numerical abilities have been reported in an Amazonian population (Mundurukù) that proved able to discriminate between large quantities even though they lack words for numbers beyond five (Pica et al., 2004). Even after being trained for many years in formal mathematics, the adults of Western societies continue to use these abilities for solving many tasks of everyday life, such as estimating the number of people in a queue or number of food items in a plate. These abilities are also supposed to be involved in laboratory studies in which participants are required to estimate which group is more numerous (e.g., 8 vs. 9 dots) in a short amount of time (e.g., 200 ms), an experimental condition that makes extremely difficult the use of verbal counting (Halberda et al., 2008).

A common finding of experiments with adults and infants is that performance in relative numerosity judgments decreases as the numerical ratio between the smaller and the larger group increases. For instance, adult humans and infants are better at discriminating 8 vs. 16 items (0.50 ratio) than 8 vs. 12 items (0.67 ratio) (e.g., Xu and Spelke, 2000; Agrillo et al., 2012). However, this ‘ratio dependence’ does not appear to be universal and in particular several authors have reported that the performance in the range 1–4 is often insensitive to numerical ratio (e.g., Feigenson et al., 2004; Revkin et al., 2008; Agrillo et al., 2013). In other words, performance seems to be very similar when discriminating 1 vs. 4 or 3 vs. 4 objects, whereas accuracy is much higher when discriminating 5 from 20 objects (1:4 ratio) than 15 from 20 objects (3:4 ratio, Atkinson et al., 1976; Oyama et al., 1981; Trick and Pylyshyn, 1993; Revkin et al., 2008). The rapid and accurate enumeration of small sets is commonly referred as ‘subitizing’, a phenomenon widely investigated in the last century (e.g., Jensen et al., 1950; Mandler and Shebo, 1982; Revkin et al., 2008).

The behavioral difference between small (≤4) and large (>4) numbers has suggested the existence of two different pre-verbal numerical systems: one precise but operating only on small numbers and one approximate, with virtually no upper limit but whose discriminability is constrained by a ratio distance (Feigenson et al., 2004; Cordes and Brannon, 2008).

To date, the exact cognitive system underlying subitizing is unknown. Several authors believe that this phenomenon is based on a process linked to a system for representing and tracking individual objects (Trick and Pylyshyn, 1994; Feigenson et al., 2002). Since this object-tracking system (OTS) keeps track of individual elements, it would be precise but would allow for the parallel representation of a small number of objects (Pylyshyn and Storm, 1988). In support of this link between human ‘subitizing’ and OTS, it has been noted that the typical subitizing range of 3–4 (Trick and Pylyshyn, 1994; Revkin et al., 2008) parallels the tracking limit of 4 (Pylyshyn, 1989). The second mechanism is an approximate number system (ANS) for representing larger numerosities: this system encodes approximate numerosities as (compressed) analog magnitudes and it supports numerosity estimation (Dehaene et al., 2003).

Subitizing and numerical estimation of large numbers appear to differ in many respects, such as speed, accuracy and cognitive load (Kaufman et al., 1949; Mandler and Shebo, 1982; Logie and Baddeley, 1987; Burr et al., 2010; Vetter et al., 2011). As noted in literature, only the ANS would be indexed by a ratio effect in numerosity comparisons (Trick and Pylyshyn, 1994; Xu and Arriaga, 2007; Revkin et al., 2008).

There is also dissociation in human ontogeny. Newborns can discriminate 2 vs. 3 but not 4 vs. 6 items in a habituation task, showing a different ratio-dependence in the two numerical ranges (Starkey and Cooper, 1980). A study of event-related potentials has provided the first neurophysiological evidence in the infant brain for different neural responses to small and large numbers (Hyde and Spelke, 2011). The authors recorded event-related potentials of 6–7.5-month-old infants in the presence of arrays containing either small (1–3) or large (8–32) sets of dots. Large numbers evoked a mid-latency parietal response (P500) that was dependent on the numerical ratio. On the contrary, small numbers evoked an earlier peaking occipital-temporal response (P400) that was dependent on the cardinal value and was not influenced by the numerical ratio.

Other studies, however, report ratio sensitivity in the small number range, challenging the idea of different mechanisms for small and large numbers. For instance, participants required to order two numerical values presented simultaneously showed ratio dependence across the range of values from 2 through 30 in accuracy and reaction time (Cantlon and Brannon, 2006). Developmental psychologists also reported data supporting one general mechanism for small and large numbers. For instance, 3-year-old children were asked to match a sample stimulus to one of two alternatives and a significant effect of numerical ratio was found in discrimination of quantities 1–4, similar to the ratio dependence reported with large sets (Cantlon et al., 2010).

To summarize, while the existence of the ANS is generally accepted and supported by the universal ratio dependence reported in large number discrimination, researchers tend to disagree as to whether a distinct precise system operates for 3–4 objects. This debate has been recently enlarged to encompass non-human species. Some authors found evidence of a different ratio dependence of the performance within and outside the subitizing range in mammals, birds and fish (Hauser et al., 2000; Hunt et al., 2008; Bonanni et al., 2011; Gòmez-Laplaza and Gerlai, 2011; Agrillo et al., 2012), while other studies report a similar ratio sensitivity in the two numerical ranges, supporting the idea of a single ANS (Cantlon and Brannon, 2007; Ward and Smuts, 2007; Tomonaga, 2008; Al Aïn et al., 2009).

It has proved difficult to resolve this issue with neuroimaging studies. If observers always *try* to subitize whatever the number of objects in the display, then there will be no distinctive signal locus for stimuli in the subitizing range (Piazza et al., 2002). One attempt to resolve this was to examine the time-course and amplitude of the BOLD signal for stimuli within and beyond the subitizing range (Piazza et al., 2003). This revealed that larger numbers elicited a larger signal than smaller numbers in the intra-parietal sulcus (IPS). By using a dual-task paradigm, Vetter et al. (2011) manipulated attentional load in a numerical task requiring them to assess whether the array contained one, three, five, or seven targets. Participants were less accurate under high load compared to low load (or single task). No interaction between load condition and target number was found, meaning that the presence of a concurrent task had the same impact in estimating small (1–3) and large (5–7) numbers. However, the authors showed that manipulating attentional load modulated the neural signal specifically in the subitizing range, and moreover, in the right temporo-parietal junction, rather than in the IPS, the locus for larger number estimation and discrimination (e.g., Piazza et al., 2003). This study shows that far from being ‘pre-attentive,’ subitizing would be an attention-demanding process (see also Burr et al., 2010). More recent psychophysiological and fNIRS studies also suggest that the processes underlying small and large number estimation are neurally dissociable (Vuokko et al., 2013; Cutini et al., 2014).

The question therefore remains as to what determines different ratio sensitivity in the small number range and, strictly related to this issue, what triggers the supposed subitizing response. Part of the problem is due to the fact that different studies use very different experimental paradigms that are likely to have a different impact on memory and cognitive load. For instance, in some tasks stimuli are simultaneously presented (e.g., Halberda et al., 2008), while in others stimuli are presented sequentially and hence subjects have to remember the numerosity of the former set (e.g., Ansari et al., 2007). Also, in some tasks distractors were introduced – thus increasing the cognitive load (e.g., Vetter et al., 2008) – while in others subjects have to solve the numerical task without distractors (e.g., Revkin et al., 2008).

To explain the inconsistency reported in the literature, it has been recently hypothesized that the ANS may sometimes be recruited to represent numbers in the subitizing range and the context in which the representation is elicited may determine which of them is employed in the subitizing range (Cordes and Brannon, 2008; Bisazza et al., 2010). If activation of the ANS in range 1–4 depends on contextual variables, such as the type of stimuli used, the method of presentation or the sensory modality involved, contrasting results may arise if different studies adopt different methods. The type of stimuli and the methodology adopted, for instance, seem to be able to affect even the range of subitizing itself. Haladjian and Pylyshyn (2011) asked participants to indicate locations of each item in a set of 2–9 disks that were displayed briefly and masked: participants proved able to attend and recall up to six items, in contrast with the four-item limit typically found when using standard reporting methods which often require to indicate how many items were presented (Mandler and Shebo, 1982). Also, it was recently demonstrated that the experimental procedure modulates the ratio dependence of children’s numerosity judgments for large numbers (Odic et al., 2014). The performance of children significantly changed depending on whether the previous history of numerical decisions was made with high- or low-confidence (experimentally manipulated by presenting to two groups of children either easier or harder discriminations and providing them with positive/negative feedback of their performance). If ratio dependence in the ANS range is affected by the history of previous trials, it is possible that different contextual factors may explain also the heterogeneous pattern of data of ratio dependence in the subitizing range.

Here we tested the hypothesis that the task context – specifically, the type of stimulus presentation – could play a decisive role in the different ratio effect observed in literature in the small number range. To this end, we adopted three of the most common procedures for investigating numerosity discrimination: simultaneous presentation of intermingled elements (e.g., Castelli et al., 2006; Halberda et al., 2008), simultaneous presentation of separate groups (Hurewitz et al., 2006; Piazza et al., 2010; Sasanguie et al., 2012, 2014) and sequential presentation of separate groups (Shuman and Kanwisher, 2004; Ansari et al., 2007).

## Experiment 1: Simultaneous Presentation Of Intermingled Arrays

### Participants

Twenty undergraduate students with a mean age of 23.3 years (range = 20 years to 27, five males) participated. The experiment was carried out at the Department of General Psychology, University of Padova. All participants were selected with normal or corrected vision.

All the experiments were approved by the ethics committee of the Department of General Psychology of University of Padova. Subjects gave written informed consent in accordance with the Declaration of Helsinki.

### Stimuli and Procedure

Stimuli consisted on 240 arrays of intermixed yellow and blue dots and appeared on a gray background. Half of the trials presented numerical contrasts with numerosities in the range 1–4 and half with numerosities in the range 6–24. In half of the trials the larger set was composed by the yellow dots, in the other half it was composed by the blue ones (diameter: 0.3–0.7 cm). We presented the same five numerical ratios, 0.25, 0.33, 0.50, 0.67, 0.75, in range 1–4 and in range 6–24. In the former the numerical contrasts were respectively 1 vs. 4, 1 vs. 3, 1 vs. 2 or 2 vs. 4, 2 vs. 3, 3 vs. 4; in the latter the numerical contrasts were 6 vs. 24, 6 vs. 18 or 7 vs. 21, 6 vs. 12 or 10 vs. 20, 6 vs. 9 or 12 vs. 18, 6 vs. 8 or 12 vs. 16. Yellow and blue dots were randomly placed in the display, therefore continuous variables (see Gebuis and Reynvoet, 2012a,b) such as density, inter-dot distance, total envelope size of each set could not be used to select the larger number of dots (minimum spacing between items: 0.4 cm). Half of the trials were controlled for cumulative surface area. In particular, we matched the total number of blue and yellow pixels, such that the total cumulative area of the two sets was identical. However, when controlling for cumulative surface area, smaller than average elements become more frequent in the more numerous sets and participants could have used this continuous cue instead of number. As a consequence, in the second half of the trials, stimuli were controlled for dot size (dots have different sizes but on the average their size was identical within each pair). Dot sizes and cumulative surface area were controlled by using TpsDig software (Rohlf, 2004), software previously used in numerical cognition studies of human and non-human species (Agrillo et al., 2008, 2010).

Stimuli were displayed on a 17-inch monitor at a comfortable viewing distance (60 cm), using E-Prime software. The experiment was conducted in a quiet and dimly illuminated room. Participants were presented with three blocks of 80 trials each. All stimuli were randomly presented within each block. The test was preceded by a short familiarization block of 20 trials with feedback.

**FIGURE 1:**
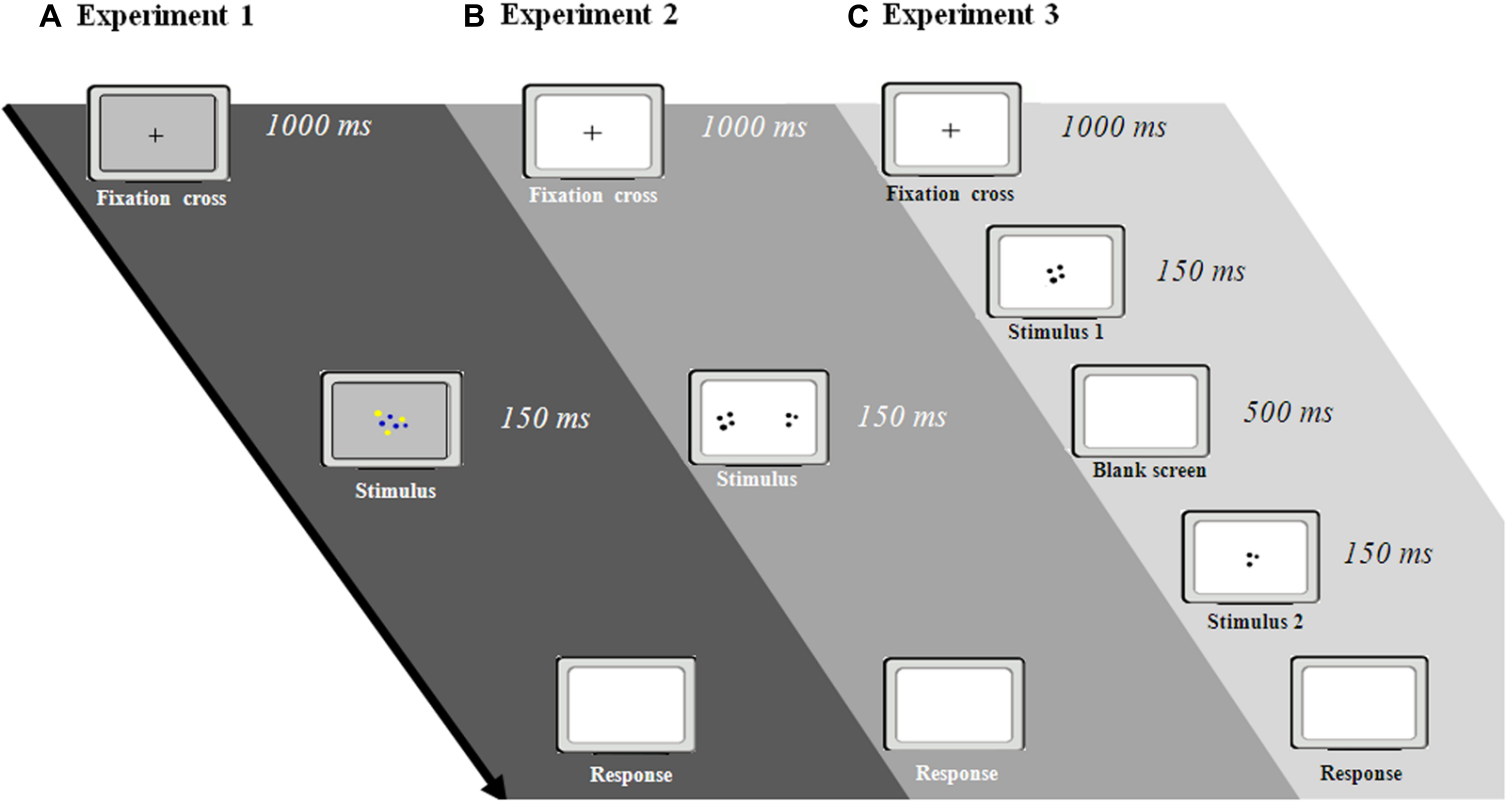
**Procedures adopted in Experiments 1–3.** Participants were required to estimate which array is more numerous in three different tasks: simultaneous presentation of intermingled arrays **(A)**, simultaneous presentation of separate arrays **(B)**, and sequential presentation of separate arrays **(C)**.

To prevent verbal processing of the stimuli, participants were asked to select, as quickly as possible, whether there were more blue or more yellow dots (**Figure 1A**). Each trial began with a fixation cross in the middle of the screen (1000 ms). This was followed by an array of blue and yellow dots intermingled, displayed for 150 ms. After the array had disappeared, participants had to respond by pressing a color-coded keyboard button. To further prevent verbal counting, a verbal suppression was introduced, in this and in the following experiments, by asking participants to continuously repeat “abc” during the whole trial (from fixation cross to the response), a procedure commonly adopted in this research field (Cordes et al., 2001; Beran et al., 2006; Frank et al., 2012).

Proportion of correct choices was used as a dependent variable to assess participants’ accuracy (Gilmore et al., 2010; Agrillo and Piffer, 2012). As performance did not reach a ceiling effect, reaction times were not analyzed. However, in order to minimize the possibility that participants’ responses were determined by verbal counting, trials with reaction times greater than 2000 ms were not included in the analyses, a criterion previously used to investigate pre-verbal cognitive abilities (e.g., Bharucha and Stoeckig, 1986; Jager and Kliegel, 2008). Data were analyzed using SPSS 20.0.

### Results and Discussion

Trials with reaction times greater than 2000 ms (0.49% of participants’ responses) were discarded. A repeated measure ANOVA with Numerical Ratio (0.25/0.33/0.50/0.67/0.75) as the within-factor was performed, separately for small and large numbers.

**FIGURE 2:**
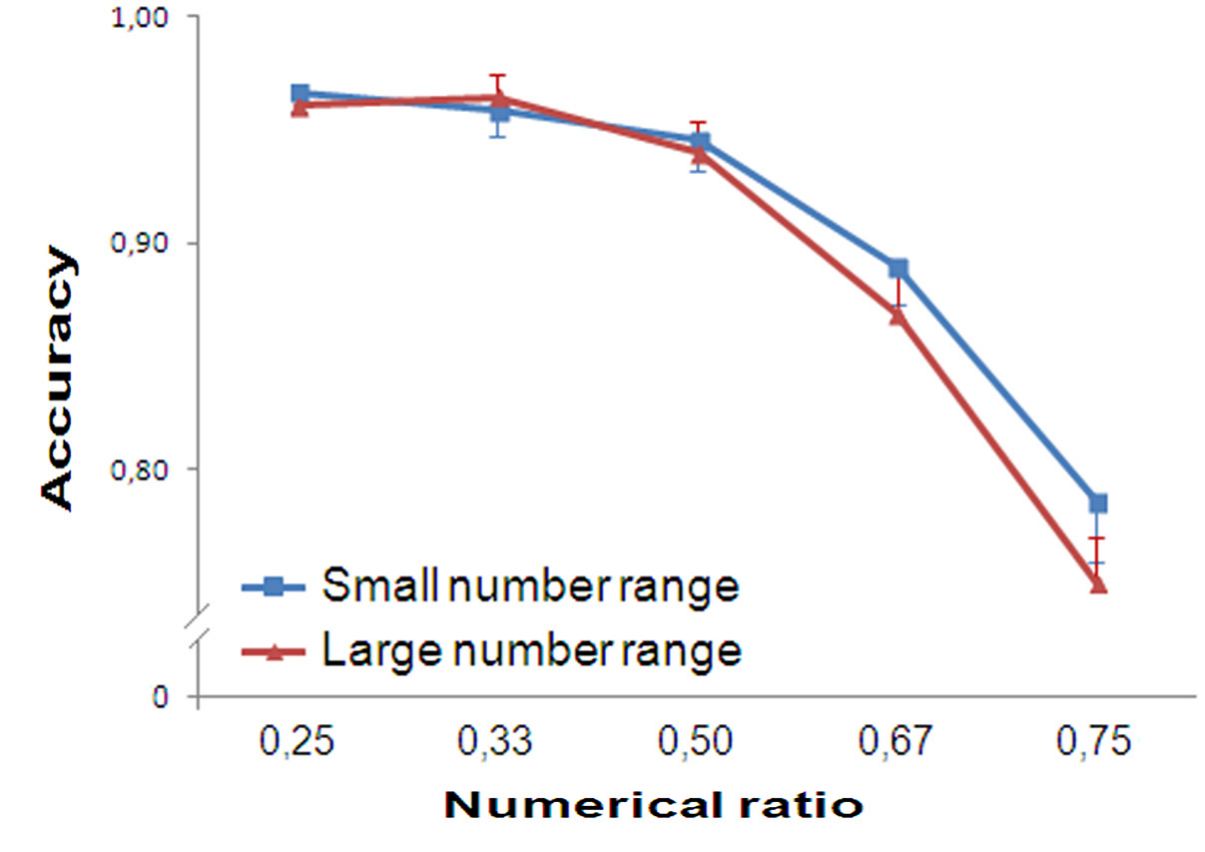
**Results of Experiment 1.** Accuracy is plotted against the numerical ratio of the contrasts for both small (1–4) and large number range (6–24). The performance of the participants showed ratio dependence for both small and large numbers. Bars in this and the following graphs represent the standard error.

In the small number range, participants’ accuracy was influenced by Numerical Ratio [*F*(4,76) = 21.755, *p* < 0.001, partial eta squared $\eta_{p}^{2}$ = 0.545]. A significant linear trend was found [*F*(1,19) = 74.726, *p* < 0.001, $\eta_{p}^{2}$ = 0.797]. In the large number range, accuracy was again affected by Numerical Ratio [*F*(4,76) = 36.320, *p* < 0.001, $\eta_{p}^{2}$ = 0.657]. A significant linear trend was also found [*F*(1,19) = 75.204, *p* < 0.001, $\eta_{p}^{2}$ = 0.798, **Figure 2**].

To assess whether the control of continuous variables is a potential factor that may affect the slopes of small and large number discrimination, we analyzed, in this and in the following experiments, whether there was a significant interaction between Continuous variable control (number + continuous variables vs. number only) and Numerical Ratio in a 2 × 5 ANOVA. No interaction was found, either in the small [*F*(4,76) = 1.478, *p* = 0.217, $\eta_{p}^{2}$ = 0.072] or in the large number range [*F*(4,76) = 1.043, *p* = 0.391, $\eta_{p}^{2}$ = 0.052].

To verify whether the slopes for small and large numbers differ statistically, we observed whether there was a significant interaction between Range (Small vs. Large) and Numerical Ratio in a 2 × 5 ANOVA. No interaction between the two factors was found [*F*(4,76) = 0.590, *p* = 0.671, $\eta_{p}^{2}$ = 0.030], meaning that numerical ratio had the same impact on the slopes of small and large number discrimination.

Lastly, in order to assess whether the salience of the stimuli was the same for yellow and blue dots, we contrasted the overall accuracy when the larger group was composed by yellow dots with that observed when the larger group was composed by the blue dots: no significant difference was found, both in the small [yellow dots: 0.913 ± 0.045; blue dots: 0.909 ± 0.043, *t*(19) = 0.495, *p* = 0.626] and in the large number range [yellow dots: 0.906 ± 0.036; blue dots: 0.906 ± 0.043, *t*(19) = 0.090, *p* = 0.929].

Participants’ accuracy in this task was affected by the numerical ratio, both for small and large numbers. As ratio-dependence did not vary as a function of the numerical range, these data are in line with the idea of a single system of numerical representation below and beyond four units. To assess whether the same results could be obtained if the arrays were spatially separated we carried out Experiment 2.

## Experiment 2: Simultaneous Presentation Of Separate Arrays

### Participants

Twenty undergraduate students with a mean age of 21.9 years (range = 19 years to 32, 4 males) participated.

### Stimuli and Procedure

We used the same numerical contrasts of Experiment 1, both for small and large numbers. Stimuli were 240 pairs of arrays of black dots of the same sizes presented as in Experiment 1 on a white background. The two arrays were displayed simultaneously, one set on the right side of the screen and the other on the left side (average distance between the two groups: 8 cm). The right–left position of the larger set was randomized. Half of the trials were controlled for continuous variables, namely cumulative surface area (summed area of dots), overall space (space encompassed by the most eccentric dots) and density of the elements (average inter-dot distance). Since density and overall space are inversely correlated, these two variables cannot be controlled simultaneously. Therefore in half of these controlled trials we equalized the overall space occupied by the arrays; in the other half of controlled trials, we equalized the density of the items. In the second half of the 240 trials stimuli were controlled for dot sizes.

As in Experiment 1, the test phase was preceded by a training phase with feedback. Each trial started with fixation cross in the center of the computer screen (1000 ms). Subsequently two arrays of dots appeared on the two sides of the screen and remained visible for 150 ms (**Figure 1B**). Participants were required to estimate whether there were more dots in the right array or in the left one, by pressing spatially congruent keys on the keyboard.

### Results and Discussion

**FIGURE 3:**
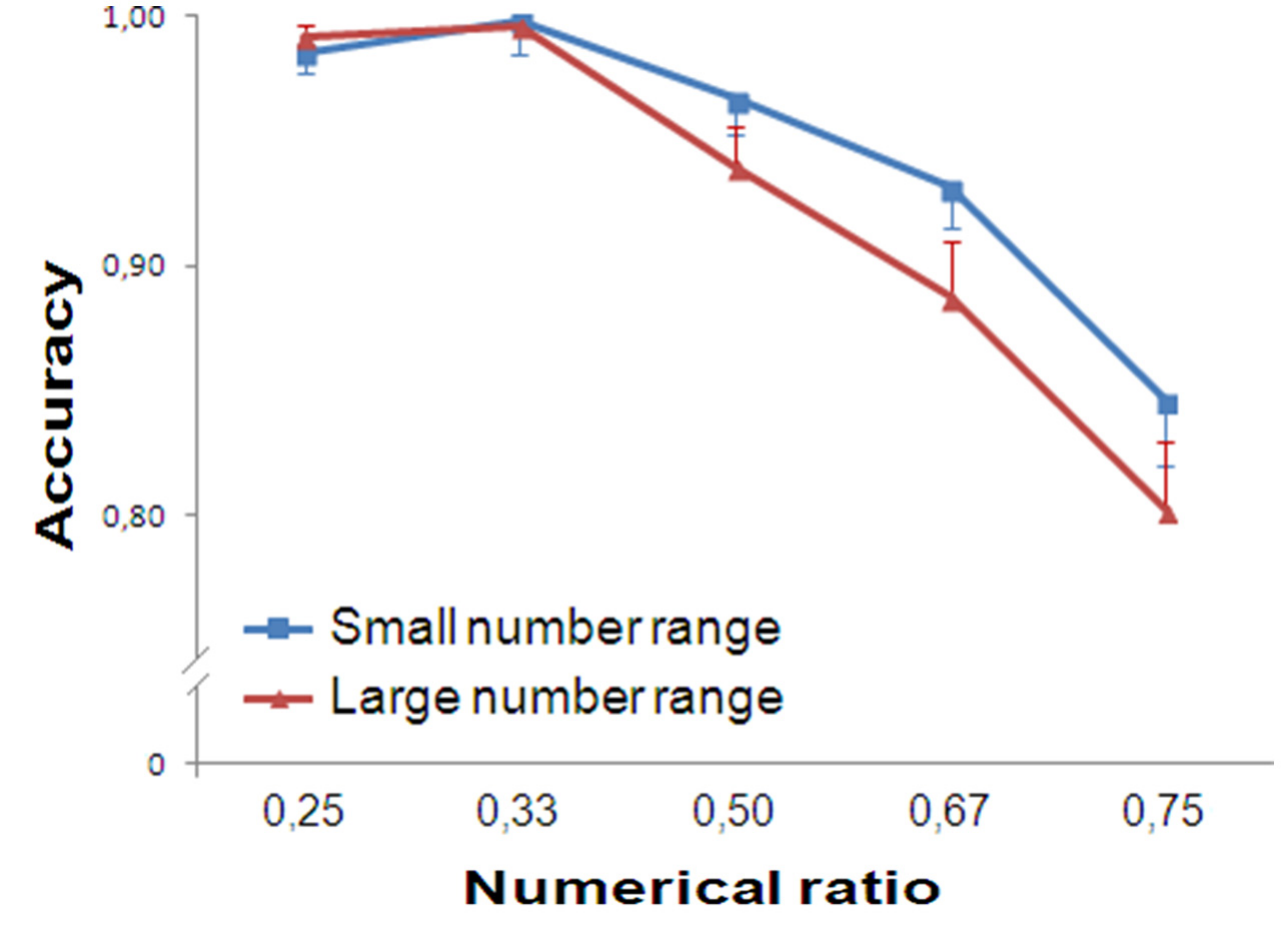
**Results of Experiment 2.** Accuracy is plotted against the numerical ratio of the contrasts for both small and large number range. The performance of the participants showed ratio dependence for both small and large numbers.

Trials with reaction times greater than 2000 ms (0.37% of participants’ responses) were discarded. As in Experiment 1, accuracy was analyzed with a repeated measure ANOVA, separately for small and large numbers. In the small number range, participants’ accuracy showed a significant main effect of Numerical Ratio [*F*(4,76) = 28.719, *p* < 0.001, $\eta_{p}^{2}$ = 0.602]. A significant linear trend was found [*F*(1,19) = 66.030, *p* < 0.001, $\eta_{p}^{2}$ = 0.777]. In the large number range, Numerical Ratio was statistically significant [*F*(4,76) = 28.276, *p* < 0.001, $\eta_{p}^{2}$ = 0.598]. A significant linear trend was found [*F*(1,19) = 51.021, *p* < 0.001, $\eta_{p}^{2}$ = 0.729, **Figure 3**].

No interaction Continuous variable control × Numerical Ratio was found, either in the small [*F*(4,76) = 1.286, *p* = 0.271, $\eta_{p}^{2}$ = 0.063] or in the large number range [*F*(4,76) = 1.067, *p* = 0.315, $\eta_{p}^{2}$ = 0.558].

No interaction Numerical Ratio × Range was found in the 2 × 5 (Range × Numerical Ratio) ANOVA [*F*(4,76) = 2.214, *p* = 0.075, $\eta_{p}^{2}$ = 0.104], meaning that the slopes of small and large number discrimination did not statistically differ.

Thus, Experiment 2 confirmed the main finding reported in the previous experiment: participants’ accuracy is set by numerical ratio both for small and large numbers. In this sense, the fact that stimuli are presented either intermingled (Experiment 1) or separately (Experiment 2) does not appear to have significant effect in terms of ratio dependence of small and large number discrimination. In both experiments, however, stimuli were simultaneously presented. In Experiment 3 we investigated whether performance can be affected by the sequential presentation of the arrays.

## Experiment 3: Sequential Presentation Of Separate Arrays

### Participants

Twenty undergraduate students with a mean age of 19.6 years (range = 19–22 years, two males) participated.

### Stimuli and Procedure

Stimuli were identical to those used in Experiment 2, with the exception that, in this test, the arrays were presented sequentially. The test phase was preceded by a training phase with feedback. Each trial started with a fixation cross for 1000 ms, followed by the presentation of an array of dots at the screen center for 150 ms. After a delay of 500 ms, another group of dots appeared for 150 ms (**Figure 1C**).

### Results and Discussion

Trials with reaction times greater than 2000 ms (0.90% of participants’ responses) were discarded. Accuracy was analyzed with a repeated measure ANOVA, separately for small and large numbers. In the small number range, participants’ accuracy was not influenced by Numerical Ratio [*F*(4,76) = 1.500, *p* = 0.211, $\eta_{p}^{2}$ = 0.073]. No significant trend was found [*F*(1,19) = 1.249, *p* = 0.278, $\eta_{p}^{2}$ = 0.062].

**FIGURE 4:**
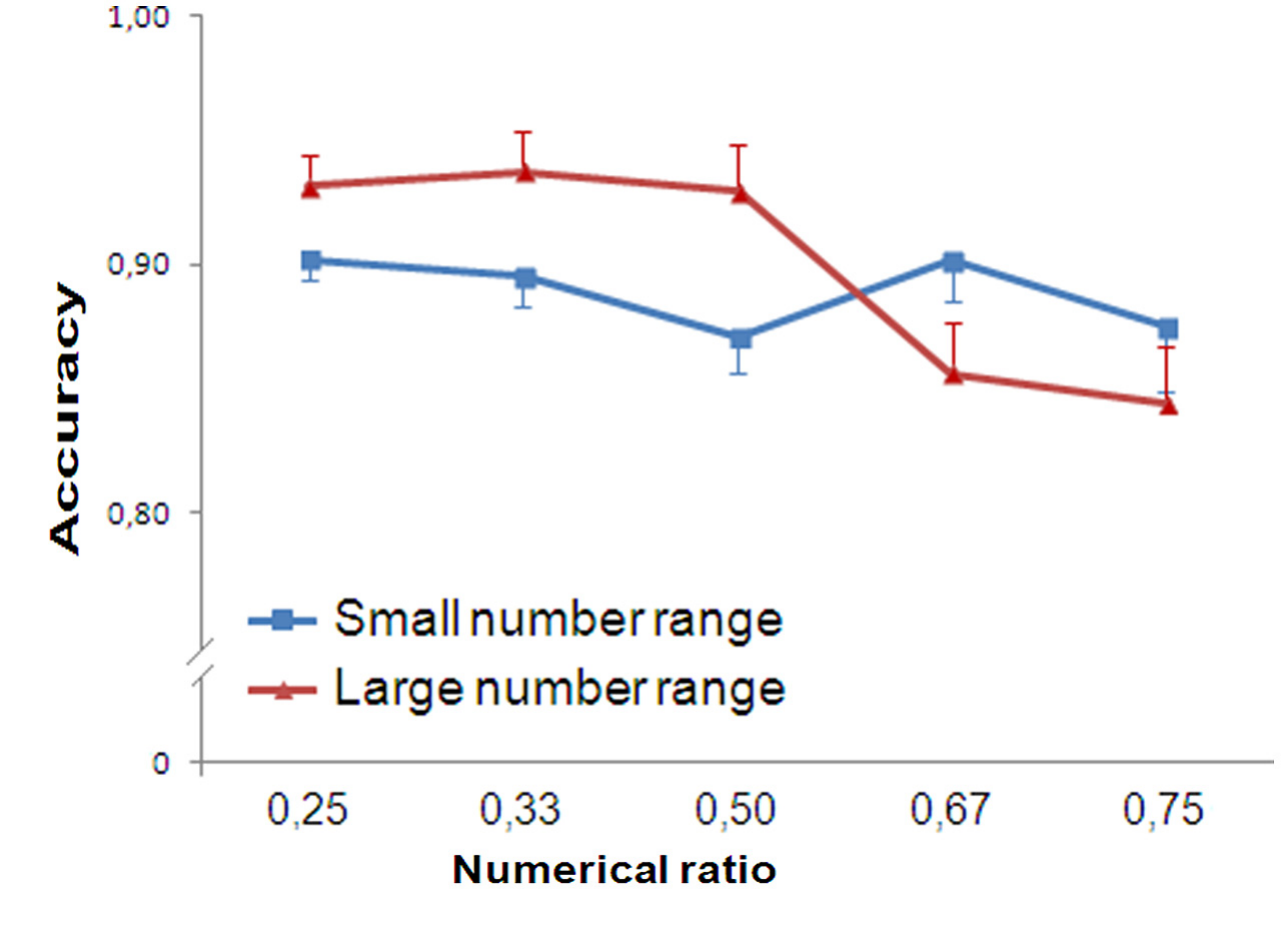
**Results of Experiment 3.** Accuracy is plotted against the numerical ratio of the contrasts for both small and large number range. The performance of the participants showed ratio dependence for large numbers and ratio insensitivity in the subitizing range.

In contrast, in the large number range, participants’ accuracy was affected by Numerical Ratio [*F*(4,76) = 18.037, *p* < 0.001, $\eta_{p}^{2}$ = 0.487]. A significant linear trend was found [*F*(1,19) = 45.496, *p* < 0.001, $\eta_{p}^{2}$ = 0.705, **Figure 4**].

No interaction Continuous variable control × Numerical Ratio was found either in the small [*F*(4,76) = 0.297, *p* = 0.592, $\eta_{p}^{2}$ = 0.015] or in the large number range [*F*(4,76) = 0.390, *p* = 0.816, $\eta_{p}^{2}$ = 0.020].

A significant interaction Range × Numerical Ratio in the 2 × 5 ANOVA (Range × Numerical Ratio) was found [*F*(4,76) = 8.132, *p* < 0.001, $\eta_{p}^{2}$ = 0.300], meaning that the slopes of small and large number discrimination statistically differ as a function of numerical ratio.

One may argue that a lack of significance in accuracy within the small number range was a consequence of a lack of power due to the small sample size. To test for this hypothesis we increased the sample size by adding 18 subjects tested in another study (Agrillo et al., 2012) that employed the same procedure. Overall in the small number range, participants’ accuracy was not influenced by Numerical Ratio [*F*(4,148) = 1.230, *p* = 0.301, $\eta_{p}^{2}$ = 0.032]. In contrast, in large number range, participants’ accuracy was affected by Numerical Ratio [*F*(4,148) = 24.353, *p* < 0.001, $\eta_{p}^{2}$ = 0.397].

Thus, participants’ accuracy is differently affected by numerical ratio for small and large numbers. As suggested by some authors (Feigenson et al., 2004; Revkin et al., 2008), the different ratio sensitivity opens to the possibility that two different cognitive systems are activated in processing small and large numbers under specific circumstances.

#### Comparison of the Three Methods

**FIGURE 5:**
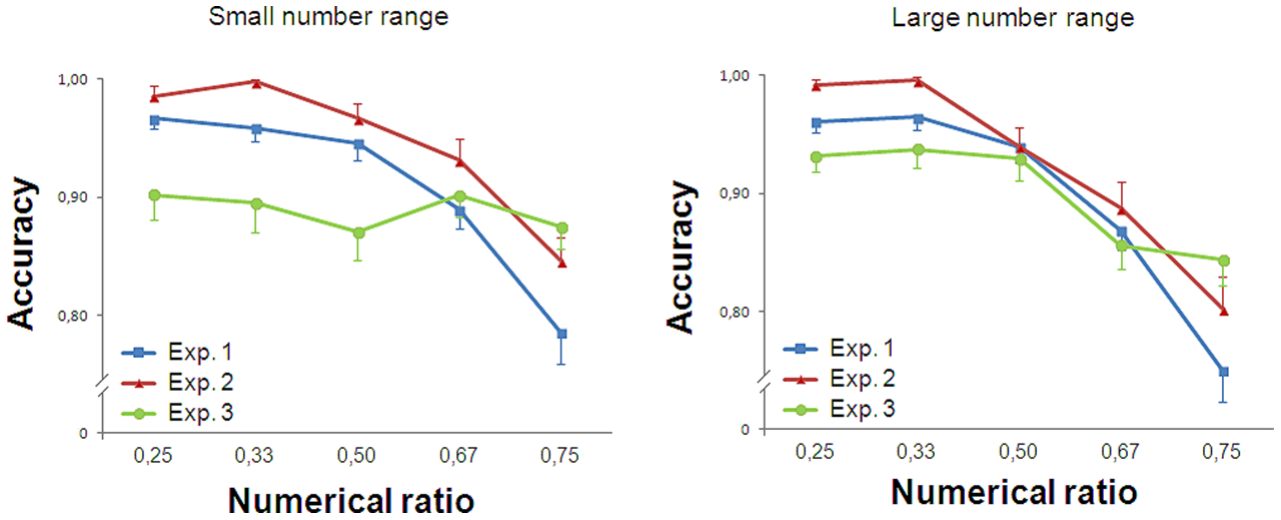
**Comparison of the three methods.** When stimuli are simultaneously presented (Experiments 1 and 2), ratio dependence of the performance was observed also in the small number range. On the contrary, when stimuli were presented sequentially (Experiment 3), participants’ accuracy did not vary as a function of numerical ratio in the small number range.

Accuracy was analyzed with a 3 (Task: simultaneous presentation of intermingled arrays/simultaneous presentation of separate arrays/sequential presentation of separate arrays) × 5 (Numerical Ratio: 0.25/0.33/0.50/0.67/0.75) ANOVA, separately for small and large numbers (**Figure 5**).

In the small number range, the accuracy was influenced by Numerical Ratio and Task [*F*(4,228) = 38.599, *p* < 0.001, $\eta_{p}^{2}$ = 0.404; *F*(2,57) = 4.632, *p* = 0.014, $\eta_{p}^{2}$ = 0.140; respectively]. The interaction between Numerical Ratio and Task was significant [*F*(8,228) = 7.856, *p* < 0.001, $\eta_{p}^{2}$ = 0.216], meaning that numerical ratio had a different impact on the slopes of the three experiments in the small number range.

In the large number range, the performance was affected by the Numerical Ratio [*F*(4,228) = 79.792, *p* < 0.001, $\eta_{p}^{2}$ = 0.583] but not Task [*F*(2,57) = 1.445, *p* = 0.244, $\eta_{p}^{2}$ = 0.048]. The interaction between Numerical Ratio and Task was significant [*F*(8,228) = 4.204, *p* < 0.001, $\eta_{p}^{2}$ = 0.129, **Figure 5**], meaning that, although in all tasks performance in the large number range is affected by numerical ratio, the impact of numerical ratio on the slopes of the three experiments is different.

To assess whether the different ratio dependence could be due to task difficulty, we performed a 2 × 3 (Numerical Ranges × Experiments) repeated measures ANOVA on the overall accuracy. Results showed a marginally significant effect of Experiments [*F*(2,57) = 3.123, *p* = 0.052, $\eta_{p}^{2}$ = 0.099]. No effect of Numerical Ranges [*F*(1,57) = 2.431, *p* = 0.125, $\eta_{p}^{2}$ = 0.041] was found. Interaction was significant [*F*(2,57) = 3.574, *p* = 0.034, $\eta_{p}^{2}$ = 0.111], meaning that participants were more accurate to estimate small numbers in Experiment 2. As participants showed ratio dependence in the small number range of this experiment, it appears unlikely that task difficulty *per se* could primarily explain the different ratio dependence reported in our experiments.

One may argue that participants seeing a single dot in the array might have used a non-numerical strategy (1 dot is always the smaller number and participants could have easily selected the second array as larger). In addition, the control of density in numerical contrasts with a single object is intrinsically problematic. Hence, we analyzed the performance of the three experiments without the two numerical ratios (0.25 and 0.33) involving the presentation of a single dot. We also excluded 1 vs. 2 contrast from the 0.50 ratio. Repeated measure ANOVA showed the same pattern of results (see **Table 1**), suggesting that the presence of a single dot did not significantly affect participants’ cognitive strategy to solve the task.

**Table 1:** Results of the three experiments in the absence of arrays composed by a single dot [only 0.50 (2 vs. 4) / 0.67 and 0.75 numerical ratios].

Experiment	Range	Ratio effect (ANOVA)	Linear trend
1	Small	*F*(2,38) = 19.064, *p* < 0.001, $\eta_{p}^{2}$ = 0.501	*F*(1,19) = 29.838, *p* < 0.001, $\eta_{p}^{2}$ = 0.611
	Large	*F*(2,38) = 33.113, *p* < 0.001, $\eta_{p}^{2}$ = 0.635	*F*(1,19) = 52.798, *p* < 0.001, $\eta_{p}^{2}$ = 0.735
2	Small	*F*(2,38) = 20.981, *p* < 0.001, $\eta_{p}^{2}$ = 0.525	*F*(1,19) = 34.751, *p* < 0.001, $\eta_{p}^{2}$ = 0.647
	Large	*F*(2,38) = 17.545, *p* < 0.001, $\eta_{p}^{2}$ = 0.480	*F*(1,19) = 36.227, *p* < 0.001, $\eta_{p}^{2}$ = 0.656
3	Small	*F*(2,38) = 2.245, *p* = 0.120, $\eta_{p}^{2}$ = 0.106	*F*(1,19) = 0.065, *p* = 0.802, $\eta_{p}^{2}$ = 0.003
	Large	*F*(2,38) = 15.347, *p* < 0.001, $\eta_{p}^{2}$ = 0.447	*F*(1,19) = 17.799, *p* < 0.001, $\eta_{p}^{2}$ = 0.484


The results from Experiments 1 and 2 show that the simultaneous presentation of the arrays (either intermingled or separate) determines a ratio effect in the small number range. This may imply that both experimental procedures automatically activate the ANS. In particular we hypothesized that a number of items exceeding the subitizing range might automatically activate the ANS even if participants were required to discriminate between small numerosities. In both experiments participants were required to segregate two sets of dots (blue from yellow dots, or right set from left set) under time constraints, which may have induced an estimation strategy. By contrast, in Experiment 3 participants saw only one group at a time, and here no ratio effect on accuracy was reported in the small number range.

As a further test of this hypothesis, we set up Experiment 4. Separate groups of dots in the range 1–4 were presented in sequence as in Experiment 3, but a group of distractors were also introduced into each array. We wanted to assess whether the total number of objects or just the task-relevant objects trigger the ANS response. Also, we investigated whether the ease of visually segregating task-relevant from task-irrelevant elements modulated the potential triggering effect of additional visual elements. If the ANS is particularly activated when the task is beyond the limit to encode items as individual objects, a ratio effect is expected in the condition that requires higher attentional resources.

## Experiment 4. Sequential Presentation Of Arrays With Distractors

### Participants

Twenty undergraduate students with a mean age of 21.81 years (range = 21 years to 26, four males) participated.

### Stimuli and Procedure

**FIGURE 6:**
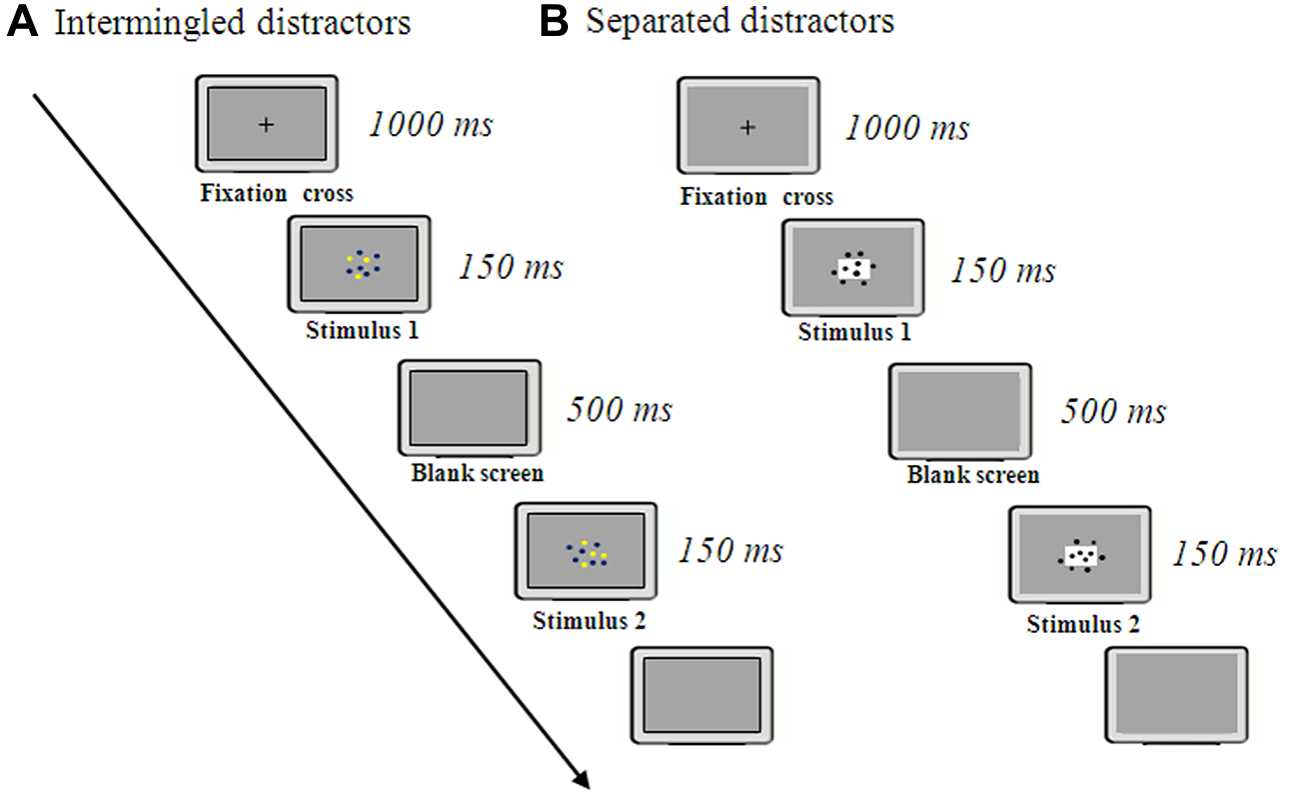
**Procedure of Experiment 4.** Participants were required to estimate the larger group when other distractors were simultaneously presented separately **(B)** or mixed together **(A)** to the target stimuli.

Target stimuli were identical to that used in Experiment 3, with the exception that, in this test, six distractors were also included within each array. Distractors were represented by dots (diameter: 0.3–0.7 cm) presented in two different ways: in the intermingled version (**Figure 6A**), each array included yellow and blue dots on a gray background, only yellow dots represented the target stimuli; in the separate version (**Figure 6B**) target stimuli were inserted on a white rectangle (3.2 cm × 5 cm) on a gray background while six other dots where placed outside the rectangle. In both versions participants were required to focus on target stimuli (yellow dots in the intermingled version, dots within the rectangle in the separate version) and have to choose the larger group. The two versions were placed in two separated blocks. In addition a control test without distractors was presented (the same condition used in Experiment 3). The three blocks were presented in a random sequence. We presented the five numerical ratios only in the range 1–4. To avoid participants using a non-numerical strategy in presence of one dot, we included also arrays including 0 target stimuli (10 trials each block). Statistical analyses, however, were performed only on the same five numerical ratios used in Experiment 1–3. As participants were not informed about the numerical range presented, participants cannot use any non-numerical strategy in the presence of 4 objects, hence numerosities larger than 4 were not presented. The test phase was preceded by a training phase with feedback. Each trial started with a fixation cross for 1000 ms, followed by the presentation of an array at the screen center for 150 ms. After a delay of 500 ms, another array appeared for 150 ms.

### Results and Discussion

Trials with reaction times greater than 2000 ms (0.46% of participants’ responses) were discarded. Accuracy was analyzed with a repeated measure ANOVA (Numerical Ratio: 0.25/0.33/0.50/0.67/0.75), separately for the type of presentation (control test with no distractors, separated arrays and arrays with intermingled distractors).

In the control set, participants’ accuracy was not influenced by Numerical Ratio [*F*(4,76) = 0.298, *p* = 0.878, $\eta_{p}^{2}$ = 0.015]. No trend was found [*F*(1,19) = 0.034, *p* = 0.856, $\eta_{p}^{2}$ = 0.02].

Similarly with the separated arrays participants’ accuracy was not influenced by Numerical Ratio [*F*(4,76) = 0.375, *p* = 0.826, $\eta_{p}^{2}$ = 0.019]. No trend was found [*F*(1,19) = 0.414, *p* = 0.528, $\eta_{p}^{2}$ = 0.021].

**FIGURE 7:**
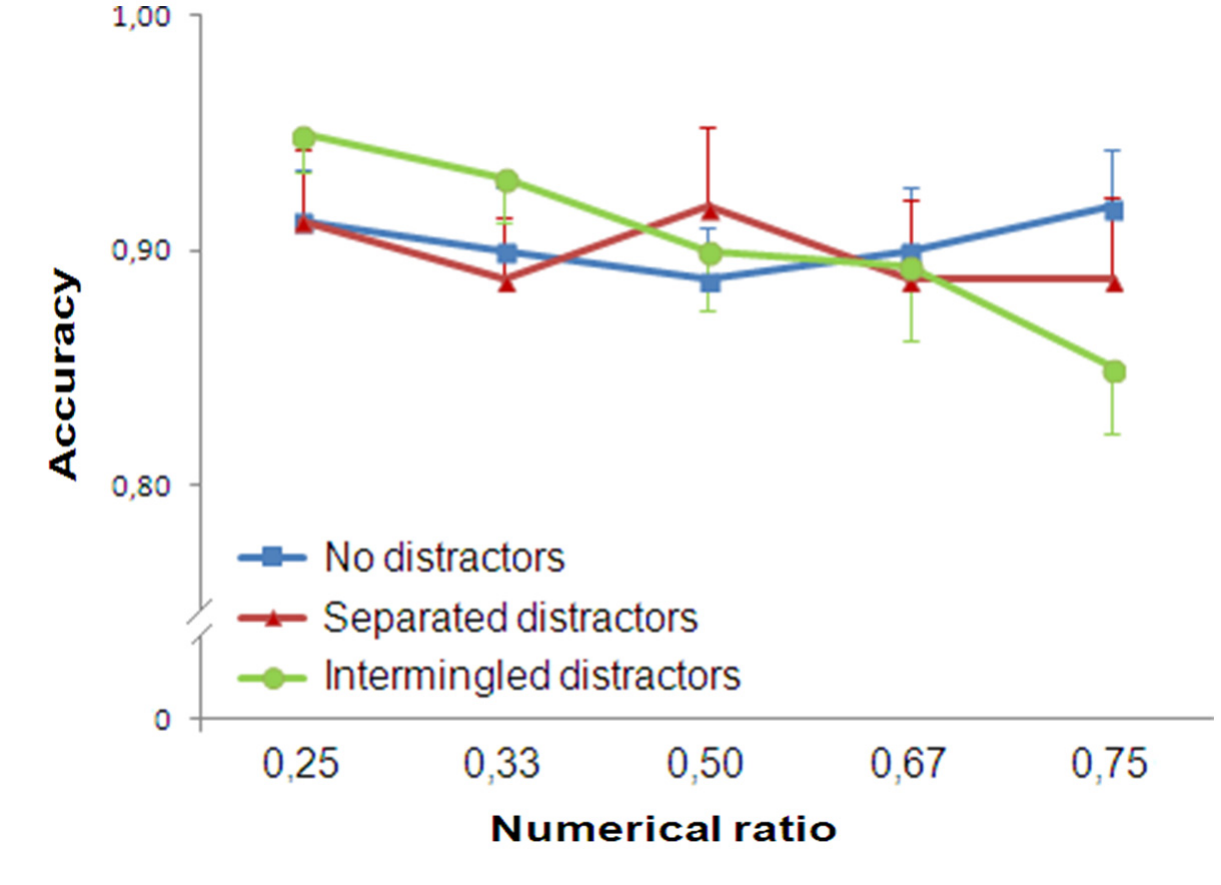
**Results of Experiment 4.** Participants’ accuracy showed ratio dependence when distractors were mixed together to the target stimuli. In contrast when no distractors were presented (control group) or when distractors were separated from the target stimuli, ratio dependence was not observed.

On the contrary, in the presence of the intermingled distractors, the participants’ accuracy was significantly affected by Numerical Ratio [*F*(4,76) = 3.687, *p* = 0.008, $\eta_{p}^{2}$ = 0.162]. A significant linear trend was found [*F*(1,19) = 20.848, *p* < 0.001, $\eta_{p}^{2}$ = 0.523; **Figure 7**].

## Experiment 5: Simultaneous Presentation Of Separate Arrays For An Extended Presentation

On the whole, results of our experiments suggest that sequential vs. simultaneous presentation of target items significantly affects ratio dependence in the small number range. However, in the sequential task (Experiment 3) stimuli were presented for 150 ms, followed by a 500 ms break, and followed by 150 ms for the second array, for a total of 800 ms processing time of the first array. The longer amount of time – compared to 150 ms used in Experiments 1 and 2 – represents a potential confound in our study. To dissociate the role of the type of presentation (simultaneous vs. sequential) or time processing of the stimuli, we set up a control experiment in which both arrays were presented simultaneously but for an extended period of time, 800 ms. If the numerical ratio continues to hold in the small number range for these extended presentations, it suggests that the format of the task, and not the presentation time, was driving the different pattern of results observed in Experiments 1–3.

### Participants

Twenty undergraduate students with a mean age of 23.5 years (range = 21 years to 26, six males) participated.

### Stimuli and Procedure

The same stimuli used in Experiment 2 were presented, both for small and large numbers. The two arrays were displayed simultaneously, one set on the right side of the screen and the other on the left side.

As in Experiment 2, the test phase was preceded by a training phase with feedback. Each trial started with fixation cross in the center of the computer screen (1000 ms). Subsequently two arrays of dots appeared on the two sides of the screen and remained visible for 800 ms. Participants (tested in a verbal suppression condition) were required to estimate whether there were more dots in the right array or in the left one, by pressing spatially congruent keys on the keyboard.

### Results and Discussion

Trials with reaction times greater than 2000 ms (0.15% of participants’ responses) were discarded. As previously, accuracy was analyzed with a repeated measure ANOVA, separately for small and large numbers.

**FIGURE 8:**
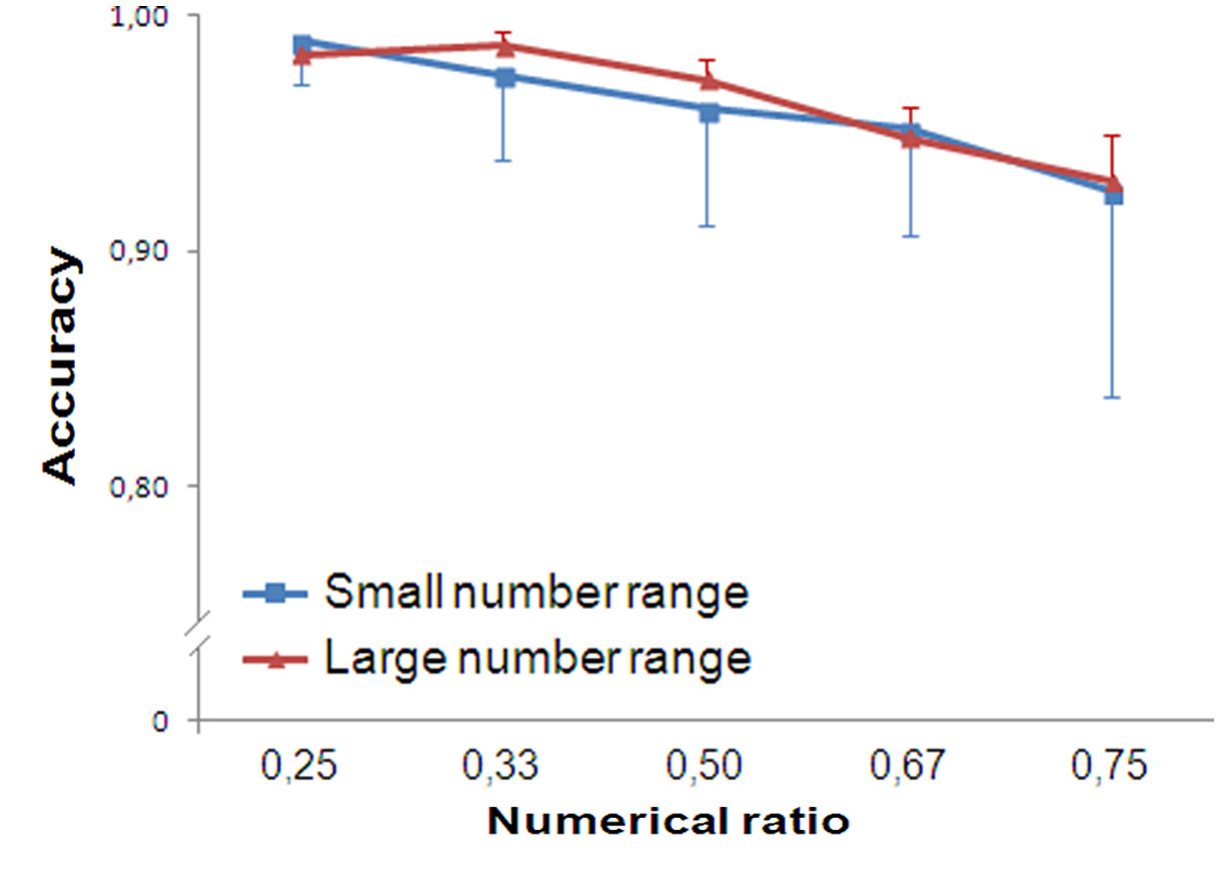
**Results of Experiment 5.** Although participants’ accuracy is close to ceiling, their performance showed ratio dependence for both small and large numbers.

In the small number range, participants’ accuracy showed a significant main effect of Numerical Ratio [*F*(4,76) = 4.700, *p* = 0.002, $\eta_{p}^{2}$ = 0.198]. A significant linear trend was found [*F*(1,19) = 11.006, *p* = 0.004, $\eta_{p}^{2}$ = 0.367]. In the large number range Numerical Ratio was statistically significant [*F*(4,76) = 5.047, *p* = 0.001, $\eta_{p}^{2}$ = 0.210]. A significant linear trend was found [*F*(1,19) = 8.781, *p* = 0.008, $\eta_{p}^{2}$ = 0.316; **Figure 8**].

No interaction Numerical Ratio × Range was found in the 2 × 5 (Range × Numerical Ratio) ANOVA [*F*(4,76) = 0.386, *p* = 0.818, $\eta_{p}^{2}$ = 0.020], meaning that the slopes of small and large number discrimination did not statistically differ as a function of the numerical ratio.

Thus, participants’ performance is close to ceiling in both numerical ranges, due to the extended presentation time. However, even in this experimental condition, numerical ratio similarly affects the accuracy of small and large numbers. It is worth noting that the two groups were simultaneously presented in comparable time conditions of Experiment 3. In this sense, we believe that the ratio insensitivity reported in the small number range of Experiment 3 cannot be due to longer presentation time, but instead is likely to be due to the format of the task (sequential stimulus presentation).

## General Conclusion

Although studies generally agree that numerical ratio affects discriminating large numerosities, they often disagree as to whether the same ratio dependence exists in the 1–4 numerical range. The exact nature of this different ratio dependence reported in literature in the small number range is currently unknown. In the present study we tested the hypothesis that the presence/absence of ratio effect in the range 1–4 might be due to the type of stimulus presentation. As the different ratio sensitivity between small and large numbers have been commonly interpreted as evidence of two separate numerical systems (OTS and ANS, Feigenson et al., 2004; Revkin et al., 2008), we supposed that the lack of ratio dependence in the small number range was indicative of activating the OTS while ratio dependence would reflect the activation of the ANS.

As predicted by the literature, the performance in the large number range showed ratio dependence with all the types of presentation. Participants became less accurate when the numerical ratio was increased in Experiments 1, 2, and 3, a condition that has been widely accepted as a clear signature of the activation of the ANS (Beran, 2004; Feigenson et al., 2004; Revkin et al., 2008; Agrillo et al., 2012). When we analyzed the performance in the small number range, we found that ratio dependence varied as a function of the type of presentation. When both arrays of stimuli were simultaneously presented (Experiments 1 and 2), ratio dependence was observed. By contrast, when the two groups were sequentially presented (Experiment 3), participants’ accuracy was not affected by the ratio. A control experiment (Experiment 5) showed that the different ratio sensitivity observed in Experiment 3 was not due to longer presentation time of the first array presented. It is worth noting that five or more objects were often presented in the visual field during Experiments 1 and 2. In those circumstances (e.g., 2 vs. 3 and 3 vs. 4) participants’ performance appeared to be worse compared to the other numerical contrasts involving a smaller number of items (e.g., 1 vs. 2). It is possible that subitizing may be affected or not existent when the total number of dots in the visual scene are larger than 4. A number of items exceeding the subitizing range may automatically activate the ANS.

Experiment 4 was devised to test this hypothesis. In two conditions a group of distractors were introduced, either separated from the target stimuli or intermingled with them. Ratio dependence was observed only when target stimuli and distractors were intermingled. Even though our data are not fully conclusive on this issue, they suggest that it is not only the total number of objects (specifically, more than four items in the visual field) that would activate the ANS, but probably a combination of different factors, such as total number of items and the ease of visually segregating task-relevant from task-irrelevant items. Indeed when target stimuli and distractors were intermingled, participants firstly had to dissociate relevant from irrelevant stimuli; on the contrary when distractors were spatially separated, focusing attention on task-relevant stimuli is supposed to be easier and presumably the task required a lower visuo-spatial working memory load.

In accordance with this interpretation, Hyde (2011) has argued that general constraints, such as attention and working memory, might determine whether a group of items is represented as individual object files in the OTS or as an approximate numerical magnitude. Usually small numbers would be represented distinctly from large numbers because they fall within our capacity to individuate and track objects simultaneously (Trick and Pylyshyn, 1994). By contrast, large numbers of objects fall outside the limits of this capacity. However, this does not exclude the possibility that small numbers of objects might also be represented approximately when the experimental procedure requires high attentional resources, such as during articulatory suppression, attentional blink, or in dual task paradigms (Hyde, 2011). Experiment 4 compared two conditions that are likely to involve different attentional resources (intermingled / higher attentional resources vs. separated distractors/lower attentional resources), and the typical signature of the ANS was observed in the condition which is supposed to be more cognitively demanding. This is consistent with the finding that attentional load can affect performance in the subitizing range (Vetter et al., 2008). This finding, incidentally, casts doubt on the idea that subitizing is pre-attentive (Trick and Pylyshyn, 1993), and is line with later findings (e.g., Burr et al., 2010).

The fact that the ANS could be activated when the task is beyond the limit to encode items as individuals objects may imply that subitizing would be the default; if the number of items (or the attentional resources) exceed a threshold, ANS would be then activated. Of course it is theoretically possible also the opposite: ANS would be the default and, if the number of items falls into the small number range, subitizing mechanisms would be recruited. However, while the one-to-one correspondence between items and object-files represents in our view a potential explanation on how to assess whether the number of items exceeds the limit of 4, it appears difficult to theorize a precise mechanism that allows the ANS to assess whether or not this limit was crossed. With respect to this topic, Chesney and Haladjian (2011) asked participants to simultaneously track objects while noticing a set of objects for enumerating and found a nice tradeoff between tracking load and subitizing performance, with a reliance on the ANS for small sets when tracking load was at maximum. A possible interpretation is that the indexing mechanism helps keep track of what “enters” the ANS (when available for smaller sets), hence reducing response variability for small sets.

Some authors have raised the question whether it is necessary to postulate the existence of two different systems on the basis simply of ratio dependence (Gallistel and Gelman, 1992; Ross, 2003). According to Gallistel and Gelman (1992) the different ratio dependence in the small and in the large number range would occur because scalar variability implies little error (noise) in the analog magnitude representations of 1, 2, 3, and 4, but increasingly more noise as the numbers get larger. Without postulating scalar variability, Ross (2003) has argued that the just noticeable difference (JND) for numerosity discrimination is about 0.25 for both small and large numerosities. All the comparisons in the subitizing range have a ratio greater or equal to 0.25. However, if Ross’s (2003) argumentation were correct, we should have found no ratio dependence in the large number range in the presence of the same ratios tested in the range 1–4, which is clearly not the case of the data reported in Experiments 1, 2, 3, and 5.

Also, some authors hypothesized that pattern recognition may be the main reason about why we often improve the performance in the small number range, reaching a ceiling effect (Neisser, 1967; Mandler and Shebo, 1982; Ashkenazi et al., 2013). In two-dimensional displays, small randomly arranged sets of objects form recognizable geometric patterns (i.e., 1 item = a dot; 2 items = a line; 3 items = a triangle, 4 items = a quadrilateral), which is not the case for random sets larger than 4 items.

Within the theoretical framework of a single system, one may argue that the results of our study may be due to a variation in task difficulty only. The harder the task, the less optimal is the performance in the small number range. We cannot entirely exclude this hypothesis, as we found a marginally significant effect of Experiments (*p* = 0.052). However, we do not feel this is the main explanation of our results, as participants showed ratio dependence in the small number range of Experiment 2, despite the fact that they exhibited a better performance in that experiment. In addition, the results of Experiment 5 showed ratio dependence in the small number range. Provided that participants had a longer presentation time of the stimuli (800 ms), it appears difficult to explain the ratio dependence observed in Experiment 5 (and not Experiment 3) just on the basis of task difficulty.

With respect to the ‘one vs. two-system’ debate, it is worth noting that data in favor of two distinct systems do not come exclusively from different ratio dependence. Indeed a potential prediction of the single-system hypothesis is that manipulation of physical properties of the stimuli should never have opposed effects on small and large number estimation, while the two-system hypothesis would allow for this possibility. Trick (2008) tested the role of item heterogeneity in the small and large number range in adult humans, finding that heterogeneity slowed enumeration in the subitizing range and sped-up enumeration in the large number range, a dissociation that is more in line with the two-system hypothesis. Moving vs. static items represent another variable that seems to affect numerical estimation differentially. As subitizing is supposed to be based on an OTS originally devoted to track and store in memory moving objects, it is supposed to be particularly activated by moving objects (Trick and Pylyshyn, 1994). Trick et al. (2003) observed that even very slow motion reduced enumeration speed for stimuli containing 6–9 items, while the enumeration of 1–4 items was not affected by moving objects. Similarly, Alston and Humphreys (2004) presented static and moving items, finding a faster and more accurate enumeration in the subitizing range in the presence of moving items. These results cannot be explained with the pattern recognition hypothesis: with moving objects, the general configuration of the arrays is continuously dynamic and no stable pattern can be recognized. Instead, the superior performance in the small number range is more in line with the idea of separate systems for small- and large-number enumeration.

In conclusion we are aware that other factors should be investigated in details before drawing firm conclusions. The absence of masking of Experiment 3, for example, was necessary to have a precise comparison with Experiments 1 and 2 in similar conditions, but in Experiment 3, we cannot exclude the possibility that an afterimage of the first array seen might have introduced a confound when comparing the three experiments. The focus of researchers should be also enlarged to encompass the effects of task paradigm. As studies on animals, infants and adults used very different stimuli – i.e., food, social companions, tones, dots (Feigenson et al., 2002; Ward and Smuts, 2007; vanMarle and Wynn, 2009; Agrillo et al., 2012) – and procedures – preferential looking time, free choice test, training procedure, sequential vs. simultaneous stimulus presentation (Castelli et al., 2006; Ansari et al., 2007; vanMarle and Wynn, 2009; Bisazza et al., 2010; Agrillo et al., 2013) it is extremely difficult to have a systematic comparison of the existing studies. Ratio dependence of the performance might appear more different than it is in human and non-human species because of very different experimental designs adopted in cognitive, developmental and comparative psychology.

The differences in ratio dependence reported here in the small number range are due to the differences in the experimental paradigms, but the debate about how many systems account our pre-verbal numerical estimation extends far beyond the aims of our work with human adults into studies of infants and the numerical abilities of non-human species.

## Conflict of Interest Statement

The authors declare that the research was conducted in the absence of any commercial or financial relationships that could be construed as a potential conflict of interest.
